# “Antimicrobial Brachytherapy” Protocol for Chronic Periprosthetic Joint Infection in Total Knee Arthroplasty: A Preliminary Case Series with 2-Year Results

**DOI:** 10.3390/jcm15114070

**Published:** 2026-05-25

**Authors:** Edward J. McPherson, Madhav Chowdhry, Adrian Lin, Alexandra I. Stavrakis, Lisa Su

**Affiliations:** 1 Department of Orthopaedic Surgery, University of California Los Angeles, Los Angeles, CA 90404, USA; alin@mednet.ucla.edu (A.L.); astavrakis@mednet.ucla.edu (A.I.S.); lsu@mednet.ucla.edu (L.S.); 2 Department of Continuing Education, Kellogg College, University of Oxford, Oxford OX1 2JD, UK

**Keywords:** Periprosthetic Joint Infection, PJI, Total Knee Arthroplasty, TKA, Antimicrobial Brachytherapy Protocol, ABP, 1.5 exchange, Antimicrobial Loaded Calcium Sulfate Beads, ALCSB

## Abstract

**Background:** The success rates of treating chronic periprosthetic joint infection (PJI) have plateaued in the last two decades utilizing exchange protocols combined with extended parenteral antimicrobials. At the same time, extended systemic antimicrobials carry well-recognized adverse effects, including organ toxicity, metabolic derangements, and disruption of host–microbiome balance, which may themselves impair host inflammatory responses and tissue healing. The defined challenge is to intensify local site eradication while minimizing systemic antimicrobial exposure and its associated host harms. This study introduces a tactical treatment shift focusing on local microbial biocidal treatment, that we aptly name “antimicrobial brachytherapy” protocol (ABP). **Methods:** A consecutive case series of 25 patients with chronic PJI in total knee arthroplasty (TKA) were treated with the ABP from 2019 to 2023. In brief, the protocol includes: (1) local multimodal antimicrobial therapy applied during debridement to reach and extinguish microbial reserves, (2) eliminating parenteral antimicrobials and limiting oral antimicrobials to 3 weeks, and (3) employing variable explantation times (longer when needed) via a 1.5 exchange technique allowing individualized host and limb rejuvenation. Patients were graded according to McPherson host scoring. Musculoskeletal Infection Society (MSIS) tier levels were used to rate success. All patients had a minimum two-year follow-up. **Results:** Eighty percent of the patients were significantly compromised, consisting of B and C hosts having 2 and 3 limb scores. Outcomes using the MSIS tier rating show 16/25 (64%) tier 1 success (infection-free without antimicrobial suppression). In these 16 patients, three were still using their 1.5 implant at a mean 4.2 years (2–5.5 years). There were two (8%) tier 3C outcomes (aseptic revision at <1 year), and six (25%) tier 3E failures (amputation, resection arthroplasty or arthrodesis) with four amputations for continued infection, and two who underwent a repeat 1.5 exchange for recurrent infection. Of the four patients amputated, three had fungal microbes identified in post-resection aspirations. Lastly, there was one (4.15%) tier 4a outcome (mortality at <1 year). **Conclusions:** The ABP concept is a tactical shift focusing antimicrobial therapy within the zone of infection, avoiding long course systemic antimicrobials, and allowing variable explantation time for host and limb rejuvenation. Success was comparable to published MSIS tier 1 outcomes using traditional two-stage exchange protocols with extended parenteral antimicrobials. Given the small sample size and absence of a comparator, the results should be regarded as hypothesis-generating, and require validation in larger, randomized, controlled studies.

## 1. Introduction

The treatment of periprosthetic joint infection (PJI) requires formidable effort and consumes a disproportionate amount of healthcare budgets [1,2]. Infection recurrence has remained steady in the last two decades despite efforts to formalize diagnosis through consensus, codifying treatment pathways, implementing aggressive debridement protocols, and using extended perioperative antimicrobial therapy [2,3,4]. Microbial adaptation is one factor implicated for the lack of improvement. In addition to antimicrobial resistance and biofilm formation, multiple additional adaptive mechanisms allow survivor advantage [5,6]. Microbial reserves persist within the periprosthetic space that can include the intracellular residence of microbes within inflammatory cells and osteocytes that includes the osteo-lacuno canalicular network (OLCN) [7]. Regional intra-bone transport creates microbial skip lesions within this network that are difficult to identify clinically [8,9,10]. Growth-arrested persister cells later reactivate when antimicrobial stresses are removed. In addition, microbes liberate cytokines interacting with host immune cells to deflect immune responses away from microbial populations. Finally, microbes deform to reach the smallest osteon spaces inaccessible to host cells [7,11,12,13,14]. The distance of microbial penetration within the periprosthetic limb space in each case is unknown.

Improving PJI success requires change, and the defined challenge is to better eradicate microbiota at the local site while maintaining systemic host health. Newer concepts include focused local antimicrobial therapy and limiting systemic antimicrobial exposure, which has been associated with organ toxicity, metabolic derangements, and disruption of host–microbiome balance that may adversely affect host inflammatory responses [15,16]. Moreover, longer explantation intervals may be needed to allow additional time in select patients for host and limb rejuvenation [17,18,19]. This study describes a PJI treatment concept that we name “antimicrobial brachytherapy” protocol (ABP), developed over the last decade. We use this phrase to emphasize the conceptual analogy to oncologic brachytherapy where local internal radiation provides targeted treatment. Similarly, ABP delivers targeted antimicrobial treatment to the infection focus, attempting to minimize systemic organ effects caused by extended systemic antimicrobials. This study describes our current exploratory, hypothesis-driven tactical strategy of ABP and reports our preliminary results treating a consecutive case series of chronic total knee arthroplasty (TKA) PJI patients.

## 2. Methods

A consecutive series of 25 ABPs was performed at a referral center from January 2019 to May 2023. Patients with chronic PJI after TKA (both primary and revision) undergoing an exchange procedure were included for study. Patients excluded were those who elected non-operative treatment. PJI was defined according to the International Consensus Meeting 2018 (ICM-18) definition [20]. All subjects had 1–2 preoperative aspirations that included microbial DNA Next Generation Sequencing (NGS) used as confirmatory data to ICM-18 criteria [21,22]. Multiple intra-operative cultures were obtained (typically 6) at resection and reimplantation. Each patient was categorized according to McPherson Staging system for rating host and limb health [23,24]. Outcomes at two-year follow-up were rated according to the Musculo-Skeletal Infection Society (MSIS) Tier Outcome Reporting Tool [4,25]. The antimicrobial brachytherapy protocol (ABP) is summarized in Table 1 with the protocol described below. Ethical review and approval were waived for this study due to its retrospective design and use of de-identified data, meeting the criteria for exemption under 45 CFR 46.104. The study was certified as exempt by the Institutional Review Board (Protocol IRB-25-2748; FWA00004642), with a waiver of HIPAA Research Authorization granted for the entire study.

### 2.1. Antimicrobial Brachytherapy Protocol (ABP)

#### 2.1.1. 1.5 Implant Exchange

**Figure 1:**
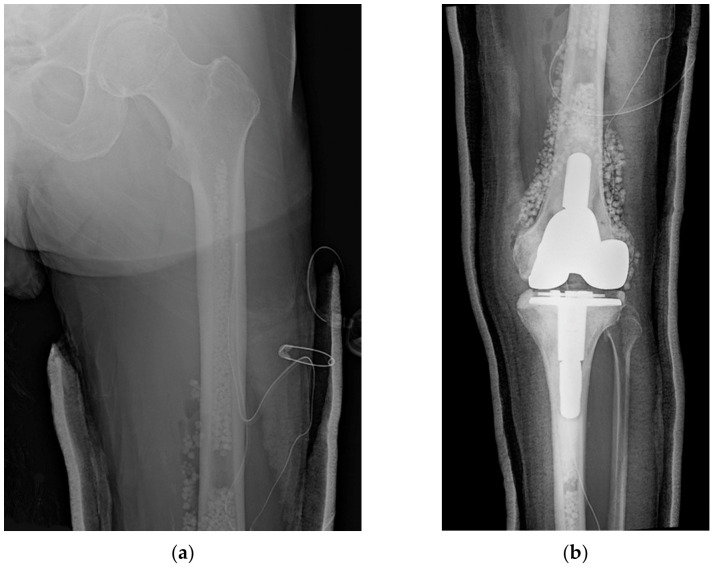

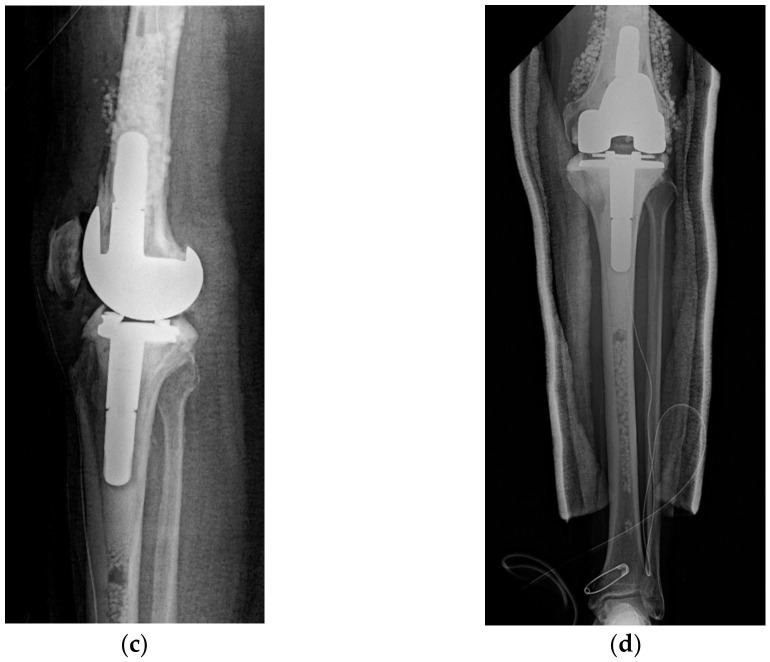
(**a**–**d**): Radiographs demonstrating articulating 1.5 exchange TKA. (**a**) Anterior–posterior (AP) view of proximal femur showing ALCSB delivered within the medullary canal using 3.0 and 4.6 mm beads. In this case 40 cc CaSO_4_ powder (≈100 cc bead volume) was delivered within the medullary canals and joint. (**b**) AP knee showing constrained revision knee implants cemented with high-dose antimicrobial loaded cement. Stems are short and smooth. Note that tibial implant is one size smaller than trial size to circumferentially coat the polyethylene insert. The periphery of the polyethylene is drilled with a 5.0 mm drill to allow the cement to interdigitate. Also note antimicrobial loaded CaSO_4_ beads filling medullary canals and compacted serving as cement restrictors. (**c**) Lateral knee view showing the patella resurfaced with an undersized implant with cement completely covering the patellar bone and polyethylene except for the apical 33% of the polyethylene dome. Note again how beads serve as a cement restrictor. (**d**) AP tibia showing ALCSB delivered within the medullary canal using 3.0 and 4.6 mm beads.

**Figure 2:**
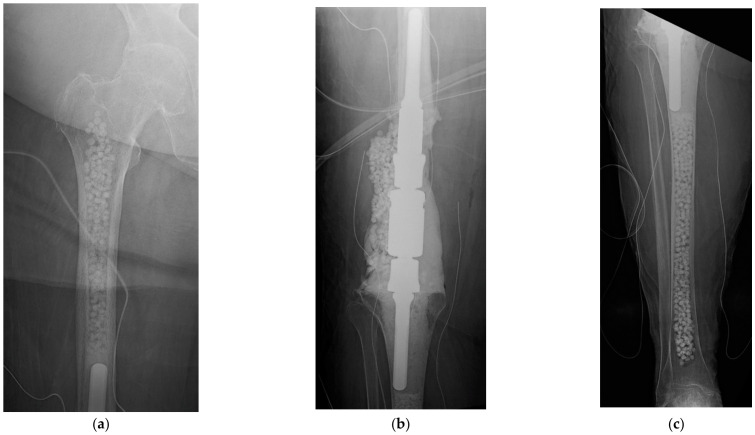
(**a**–**c**) Non-articulating 1.5 exchange modular endofusion. (**a**) AP proximal femur showing ALCSB delivered within the medullary canal using 3.0 and 4.6 mm beads. In this case 80 cc CaSO_4_ powder (≈200 cc bead volume) was delivered within the medullary canals and joint, with most bead volume dispensed within the medullary canals. (**b**) AP knee region showing modular endofusion construct. The stems are cemented first, followed by trialing to equalize limb length and adjust foot rotation. The endofusion device is connected via a clamshell device, and then completely coated with high-dose antimicrobial PMMA. (**c**) AP tibia, noting again antimicrobial loaded CaSO_4_ beads (3.0 and 4.6 mm) filling the medullary canal and acting as a cement restrictor. In the 1.5 exchange, cement is injected and manually pressurized during cementation.

In the literature, the 1.5 exchange has several interpretations [26,27]. For our protocol, the 1.5 exchange refers specifically to using commercial definitive implants (not all-acrylic cement spacers), cemented with *high*-dose antimicrobial loaded polymethylmethacrylate (PMMA). In this technique, implants are appropriately sized but inserted with short, cemented stems and no metallic support cones, to allow for extraction later when needed. This is opposed to a single-stage exchange where definitive implants are selected that are generally more extensile and are cemented with *low*-dose antimicrobial PMMA. The initial debridement surgery included resection of implants and all ancillary biomaterials. Upon completion of lavage and debridement, implants were cemented using modern cement technique, employing *high-dose* antimicrobials within PMMA. Cement mantles were purposely made thin (1–3 mm) by using large diameter short smooth stems. This allowed for easier extraction later. In articulating knee constructs, a constrained TKA implant system was used. In knees with segmental bone resection and/or global instability, a modular endofusion implant was used (Figure 1 and Figure 2). Surgical drains were removed no later than 3 days post-op. All patients were allowed full weight without restriction in activity.

#### 2.1.2. Antimicrobial Loaded Bone Cement

Palacos cement (Heraeus Medical GmbH, Hanau, DE) was mixed with an established formulation of 8.6 g of antimicrobial powder (Table 1) [28]. In each 40 g bag of cement powder, 5 g of vancomycin powder (Pfizer (Hospira), Lake Forest, IL, USA) and 3.6 g of tobramycin powder (Pfizer (Hospira), Lake Forest, IL, USA) were added and homogenized prior to adding the monomer. When needed, the antifungal voriconazole 400 mg powder (Pfizer Inc., New York, NY, USA) was added to the above antimicrobials. To allow for mixing of 8.6 g of antimicrobial powder, 10 cc of sterile saline and 1 cc of methylene blue was added along with the liquid monomer and mixed in a closed mixing container without suction [29]. The cement was inserted at 3 min using an injection gun and the cement manually pressurized.

#### 2.1.3. Antiseptic Lavage

The antiseptic lavage was a 1:1 mixture of 10% povidone iodine (McKesson, Irving, TX, USA) and 3% hydrogen peroxide (PIHP) (Owens & Minor, Mechanicsville, VA, USA) that was mixed at the point of care on the operative field [15,16,30]. Once mixed, PIHP was immediately delivered into the surgical wound and medullary canals via bulb syringe with a dwell time of 3–5 min, immediately followed by saline lavage until clear [30]. Two to three PIHP applications were administered during debridement.

#### 2.1.4. Antimicrobial Loaded Calcium Sulfate (CaSO_4_) Beads (ALCSBs)

Pharmaceutical derived CaSO_4_ powder (Synthecure, Heraeus Medical GmbH Hanau, DE) was mixed with an established formulation of antimicrobials [31,32]. In each 10 cc of CaSO_4_ powder, 1 g vancomycin powder and 240 mg of liquid (6 cc) tobramycin was mixed to a paste and spread into a silicone bead mold creating 3 mm and 4.6 mm beads and allowed to set. When needed, the antifungal voriconazole 200 mg was added to the above antimicrobials [33]. Beads were delivered to fill the medullary canals to the level of implant stems. The ALCSB served a secondary purpose as a cement restrictor. Generally, 25–35 cc bead volume was placed within the joint (note: 10 cc of CaSO_4_ powder once hydrated with saline, makes approximately 25 cc bead volume).

#### 2.1.5. Parenteral Antimicrobials

Parenteral antimicrobials were limited to no longer than three days, using a weight-based first-generation cephalosporin (Ancef, Sandoz, Basel, CH). The parenteral antimicrobial was used in the capacity as surgical prophylaxis only. In this series, there were no penicillin allergic cases.

#### 2.1.6. Oral Antimicrobials

After completion of parenteral antimicrobials, patients were started on an oral antimicrobial and continued upon hospital discharge for a total duration not to exceed 3 weeks. Doxycycline (100 mg twice daily) was the default antimicrobial as it is well tolerated, conferring good compliance with twice-a-day dosing. Further, it is human cell membrane-permeable, reaching intracellular microbial residents. However, the oral agent selected was ultimately based upon microbial sensitivity. Patients with fungal microbes were treated with an azole antimicrobial based upon sensitivity, for a period of 16–24 weeks. Antifungals were continued indefinitely in those cases with a chronic fungal source, e.g., pulmonary fungal infection.

#### 2.1.7. Serum Monitoring

Serum levels of vancomycin, tobramycin, calcium, and creatinine were monitored daily, as antimicrobial release from ALCSB and PMMA shows an initial burst followed by a steady state release (note: ALCSB within the medullary canal do not contribute to burst levels) [31,34,35]. IV hydration was started postoperatively at 125 cc/h for the first 48 h and was gradually reduced as serum antimicrobials fell below minimal detectable levels. In cases of chronic kidney disease, the antimicrobial doses remained unchanged in ALCSB and antimicrobial PMMA; however, the intra-articular bead volume was reduced to less than 20 cc. In addition, a longer period of high-volume IV hydration was used to prevent further kidney injury. This hydration regimen is empirical but consistent with published reports describing transient hypercalcemia and serum antimicrobial peaks after ALCSB and antimicrobial loaded cement implantation [36].

#### 2.1.8. Reimplantation

The interval between the 1.5 resection and second reconstruction varied and was no shorter than 3.5 months. Patients were monitored with serial aspirations, and serum c-reactive protein and albumin, with all values showing normalization. Synovial fluid was tested both for culture and microbial NGS with at least two aspirations. NGS was performed by MicroGenDX (Lubbock, TX, USA) using a validated two-tier workflow: Level 1 pathogen-specific qPCR for common PJI organisms, followed by Level 2 next-generation sequencing of the bacterial 16S rRNA gene and the fungal ITS (Internal Transcribed Spacer) region, with proprietary, curated bioinformatic taxonomic assignment [21,37]. Synovial and serum values were tabulated according to ICM-18 criteria. Reimplantation was deferred if the patient remained comfortable, was undeterred in functional activities of daily living and did not want additional surgery. At reimplantation, definitive implants were cemented with low-dose antimicrobial cement. In each 40 g bag of cement powder, 1 g of vancomycin and 1.2 g of tobramycin powder were added and homogenized prior to adding the monomer. ALCSB (using the same antimicrobial formula) were applied to medullary canals and joint with intra-articular bead volumes no greater than 25 cc. Patients whose intra-operative cultures were negative were not treated with any further antimicrobials after discharge. Parenteral antimicrobials were limited to no longer than three days, used only as surgical prophylaxis.

### 2.2. Statistical Analysis

Statistical analysis was performed using SPSS version 28.0 (IBM SPSS Statistics, Chicago, IL, USA). Categorical variables were compared using the chi-square test. Student’s *t*-test (or Welch’s *t*-test where variances differed) was used for continuous outcomes. Exploratory multivariable logistic regression was performed to examine host- and limb-related factors in relation to surgical success. We emphasize that, given a sample size of 25, all inferential analyses are descriptive and exploratory; the study is underpowered to detect small or moderate effects, and the absence of statistical significance should not be interpreted as evidence of no effect. A *p*-value of <0.05 is considered statistically significant.

**Table 1:** Items of Antimicrobial Brachytherapy Protocol (ABP).

Items	Description	Dosing	Comments
**1.5 Implant Exchange** **Technique**	Cemented Commercial, Definitive Implants with High Dose Antimicrobial Loaded Bone Cement		Provides patient comfort & longer explantation time to allow host & limb rejuvenation
**High Dose** **Antimicrobial Loaded Bone Cement (ALBC)**	High Dose Antimicrobial Formula Closed bowl mixing withmanual pressurization of cement within bone Calcium sulfate beads w/in canals serve as a restrictor	40 g Palacos powder with: 5 g vancomycin powder 3.6 g tobramycin powder 10 cc saline with 1 cc methylene blue Antifungal when needed: Voriconazole 400 mg	Saline is added with the monomer to mix all powders Methylene blue serves as cement marker & accelerant
**Operative Antiseptic Lavage - 1:1 PIHP**	Lavage (including canals) after removal of all implants and debridement 1:1 mix of povidone iodine & hydrogen peroxide	10% Povidone Iodine + 3% Hydrogen peroxide Dwell time 3–5 min with each application Rinse with saline until clear	Mixed at point of care Deliver immediately after mixing. Irrigate target areas w/bulb syringe including medullary canals.
**Antimicrobial Loaded Calcium Sulfate Beads** **(ALCSB)**	Mix antimicrobials into CaSO_4_ powder Create 3.0 & 4.6 mm beads	Synthecure 10 cc CaSO_4_ powder Mix 1 g vancomycin powder & 240 mg tobramycin liquid (6 cc) per 10 cc powder Antifungal when needed: Voriconazole 200 mg per 10 cc powder	Fill the canals with beads to the level of stem tips 25–35 cc bead volume into joint space Note: 10 cc CaSO_4_ powder once hydrated with saline makes ≈25 cc bead volume
**Limited Parenteral** **Antimicrobials**	No more than 3 days Weight-based 1st or 2nd generation cephalosporin	Ancef 1–3 g every 8 h	Used for prophylactic purposes only No long-term indwelling percutaneous catheters
**Oral Antimicrobial of limited duration**	Doxycycline, if possible (human cell membrane permeable)	Duration 3 weeks only	Tailor to microbial sensitivity

## 3. Results

The mean age of subjects was 71.3 years (range 46.5 to 93.1, standard deviation (sd) = 10.2 yr). There were 14 males and 11 females with a mean body mass index of 32.4 (range 21.4–48.6). There were three A hosts, nineteen B hosts, and three C hosts. There were four Type 1 limbs, six Type 2 limbs, and fifteen Type 3 limbs. Fifteen of 25 patients (60%) were revision TKAs with 13/15 (87%) requiring an endoprosthetic fusion device to stabilize the knee at resection. Eleven of 25 patients (44%) had a previous two-stage exchange for PJI prior to the current infection episode. Seven of 25 patients (28%) had a medial gastrocnemius rotational flap utilized for soft tissue coverage at resection. Table 2 lists the organisms identified at the time of resection, noting a wide range of microbes. The most common organisms were *Staphylococcal* species. Three of 25 patients (12%) had a polymicrobial infection, and 2/25 patients (8%) were solely fungal infections. The mean time to second stage reconstruction was 11.4 months (range 3.5–36.1 months, sd = 8.8). The mean antimicrobial load (beads + PMMA) inserted into each knee was 38.8 g (range 22.2–60.2 g). It is known from prior in vitro work that antimicrobial release from PMMA is primarily a leach effect, with approximately 8% of added antimicrobials released within the first week, with the remaining quantity entrapped within the deeper regions [38,39,40,41,42]. Based on this assumed 8% PMMA release, the estimated *effective* mean antimicrobial load (beads + PMMA) available during the first week was 9.3 g (range 5.8–13.4 g) [38,40,41,42,43,44]. We emphasize that this “effective load” is an estimate extrapolated from published elution studies and was not directly measured in the present cohort. It should be interpreted as an illustrative (not quantitative) parameter.

At minimum two-year follow-up, outcomes using the MSIS tier rating showed 16 of 25 patients (64% with 95% CI 44.5–79.8%) with tier 1 success (infection-free without antimicrobial suppression). Among these 16 patients, three remained on their 1.5 implant at a mean 4.2 years (2–5.5 years) and were monitored with serial aspirations. There were two of 25 patients (8% with 95% CI 2.2–25.0%) with tier 3C outcomes (aseptic revision at 3 and 5 months), six of 25 patients (25% with 95% CI 11.5–43.4%) with tier 3E failures (amputation, resection arthroplasty or arthrodesis), including four amputations for continued infection and two repeat 1.5 exchanges for recurrent infection. Of the four patients amputated, three had fungal microbes identified in post-resection aspirations. Lastly, there was one of 25 patients (4.2% with 95% CI 0.7–19.5%) with a tier 4a outcome (mortality at 8 weeks).

The combined host and limb rating and MSIS tier 1 success rates are shown in Table 3 and Table 4. The majority of patients were significantly compromised with B and C hosts having 2 and 3 limb scores comprising 80% of the study cohort. Host grade was not significantly associated with tier 1 success (chi-square = 3.22, *p* = 0.20), and limb score was not significantly associated with tier 1 success (chi-square = 2.60, *p* = 0.27). Exploratory multivariable logistic regression did not identify significant independent associations for host grade or limb score. As described in the Statistical Analysis section, these comparisons should be interpreted descriptively only; given the sample size of 25, the study is underpowered to detect small or moderate effects, and the absence of statistical significance should not be interpreted as evidence of no association.

In patients without a prior history of two-stage exchange, tier one success rate was achieved in 10 of 14 patients (71.4%). In comparison, in patients with a prior history of a two-stage exchange, the tier one success rate was six out of 11 (54.4%), which was not statistically significant (chi-square = 0.76, *p* = 0.38). Mean CaSO_4_ bead powder in those with tier 1 success versus all remaining tier groups was comparable, with 48 cc (range 30–80 cc, sd = 12.7) versus 51 cc (range 30–70 cc, sd = 10.5), respectively (Welch t = −0.63, *p* = 0.53). The *effective* mean antimicrobial loads inserted within each knee (beads + PMMA) were comparable, with those with tier 1 success at 9.1 g (range 5.8–13.4 g) versus all remaining tier groups at 9.6 g (range 6.5–12.7 g), Welch’s t = −0.71, *p* = 0.48.

## 4. Discussion

The treatment of chronic PJI is at a crossroads. Despite efforts to improve outcomes, success rates in the last two decades have plateaued, utilizing exchange protocols combined with extended systemic antimicrobials [2,3,4,45,46]. There is growing opinion of changing the treatment paradigm for PJI [45,47,48]. We believe that therapeutic improvement requires tactical innovation, for which local site multimodal antimicrobial treatment is of novel focus [31,32,33,49]. There is growing knowledge of the detrimental effects of extended systemic antimicrobials affecting host–microbiome balance, creating dysbiosis [50]. Disruption of this balance has profound downstream effects on cytokine signaling, inflammation, and tissue repair throughout the body [51,52,53,54,55,56,57,58]. At the blood–joint barrier, dysbiosis-induced pro-inflammatory cytokines alter permeability, increasing risk for microbial recolonization [56,57,58,59]. Moreover, parenteral antimicrobials, through multiple mechanisms, adversely affect human cells causing organ dysfunction [60]. If antimicrobial treatment locally can achieve similar microbial bioload reductions, avoiding extended systemic antimicrobials may provide host advantage. Lastly, standard exchange intervals (typically ≤12 weeks) may be inadequate for physiologic recovery in all patients, with some requiring longer intervals to rejuvenate host and limb health [61,62], emphasizing that the host immune system ultimately is required to clear an infection and maintain an infection-free environment. The ABP addresses these concerns. We believe that the balance of benefit versus harm from extended systemic parenteral antimicrobials in chronic PJI is increasingly being questioned in the literature, and ABP is offered as one investigational alternative. The elemental concepts of ABP are: (1) applying local site, multimodal antimicrobial therapy during debridement to reach and extinguish microbial variant reserves, (2) eliminating parenteral antimicrobials and limiting oral antimicrobials to 3 weeks, and (3) employing variable explantation times (longer when needed) via a 1.5 exchange technique allowing individualized host and limb rejuvenation.

Typical of a specialized referral service, this series was skewed toward substantial host and limb compromise. Eighty-eight percent were B/C hosts and sixty percent were limb score 3. Despite this, tier 1 success of this cohort was 64%. For first time infections, our tier 1 success was 71.4%. Our current ABP shows results comparable to traditional exchange algorithms in THA and TKA with a reported tier 1 range of 38–64% [4,25,63,64,65,66]. Further, we believe that tier 1 success is harder to achieve in TKA compared to THA due to the limited soft tissue coverage at the knee. In the literature review, the MSIS tier 1 success of 2-stage exchange TKA is reported between 54 and 62.5% [25,63,64,65,66]. Comparatively, our tier 1 success is noteworthy in consideration of the significantly compromised study cohort, the high number of infected revisions, and avoiding long-term systemic antimicrobials. Our preliminary findings are in concordance with emerging results of the Short or Long Antibiotic Regimes in Orthopaedics (SOLARIO) trial which supports shortened systemic antimicrobial therapy when combined with local antimicrobial delivery for PJI [67].

The amputation rate of 16% in this series warrants contextual interpretation. Our cohort was heavily weighted toward severely compromised limbs, with 15 of 25 patients (60%) graded as limb score 3, the most compromised category in the validated McPherson PJI staging system [24]. This study reported a 25% amputation rate in limb score 3 patients, which would project 6.25 amputations in a cohort of our size and composition. Our observed result of four amputations (16%) is therefore lower than the historical benchmark for this limb-grade distribution. Additionally, three of the four amputations in our series occurred in patients with fungal PJI, an entity not represented in the original 1999 cohort. Fungal PJI is widely recognized as more refractory than bacterial PJI, with published amputation rates of 15–40% [68,69]. Together, these findings support the interpretation that limb-grade severity and fungal pathogen status are the dominant drivers of amputation risk in this cohort. Lastly, although our cohort size is small, our case series results are encouraging and serve as a reference pilot study for future rigorous investigation of the brachytherapy concept in larger randomized trials.

We believe that the ABP imparts a therapeutic advantage for multiple reasons. First, local multimodal antimicrobial treatment is synergistic spanning the perioperative period. The application of non-specific PIHP antiseptic reaches into medullary canals, micro-osteons and potentially the OLCN interstices, providing immediate non-specific microbial kill. Recent in vitro studies have shown PIHP to be the most effective mixture in killing microbes in both planktonic and biofilm states, including fungi and polymicrobial conditions [16,30,70,71]. Additionally, PIHP disrupts host cells, allowing intra-cellular microbial variants to be liberated and extinguished. High-dose antimicrobial PMMA elutes antimicrobials to adjacent implant–bone interfaces and joint. Antimicrobial loaded CaSO_4_ beads are important for its acute and intermediate-term release of antimicrobials, potentially exhausting remaining microbial reserves, including potential intra-bone skip lesions. A large joint in vitro model has shown high levels of vancomycin (above biofilm killing doses) eluting past six weeks and tobramycin eluting up to 3 weeks, which at these levels can prevent biofilm reformation on implants, and permeate the osteonal microarchitecture to reach microbes residing within micro-osteonal spaces [31,72]. At our center, the dual antimicrobial formula in CaSO_4_ beads has always been vancomycin and tobramycin. This combination provides broad-spectrum antimicrobial coverage, but more importantly provides synergistic microbial kill via their mechanisms affecting microbial disruption [73,74,75]. With ALCSB elution showing antimicrobial levels far exceeding minimum bactericidal concentration (mbc) levels, this combination is potentially effective against a greater spectrum of encountered microbes than what is suggested on antimicrobial susceptibility panels. Further, we propose an additional speculative antimicrobial mechanism involving ALCSB via a pH shift. Vancomycin has an inherent pH of 3.5 which creates an acidic fluid environment [31,76]. With extended release of vancomycin from CaSO_4_ beads, we hypothesize that the local wound is acidified, despite attempted host buffering, thereby challenging microbial survival. A low-pH environment is known to kill microbes, but this proposed mechanism requires in vitro and in vivo verification using ALCSB [77,78]. The entirety of mechanisms discussed above may explain our tier 1 success with ABP despite using only one antimicrobial combination in all cases. Our results may be a clue in providing a tactical adjustment away from employing “germ-specific” antimicrobials, towards non-specific antiseptic agents. Further study of this concept is required.

Second, by limiting parenteral antimicrobial therapy, there is potentially less disruption to host–microbiome balance. The disruption of host microbiomes has been associated with adverse downstream consequences that include impaired nutrition, healing, neurotransmitter communication, and the activation of microbial resistance with adaptive genes that can pass from gut to the affected joint [79,80,81,82]. Studies have shown that disruption of the host–microbiome balance with long-term systemic antimicrobials is known to increase morbidity and mortality [83]. Lastly, we feel strongly that the time element of interval resection requires change. Many patients afflicted with PJI have some aspect of compromise, either host weakness, limb tissue damage or both. It is known that the blood synovial barrier requires at least 3 months to reconstitute, and its protective integrity takes even longer to reestablish, as pro-inflammatory markers recede [84,85]. Two-stage exchange protocols all describe rest intervals ranging from 4 to 16 weeks, but not all patients recuperate in similar [61,62]. We believe that recovery of host and limb health is variable with some patients requiring longer explantation intervals. Although treatment for PJI significantly reduces microbial load, a rejuvenated immune system is essential for complete eradication of the infection and maintenance of an infection-free environment. This is reflected in the past literature where longer explantation intervals of up to one year showed better survivorship in exchange protocols [61,62]. Our 1.5 exchange technique provides the added time when needed. The 1.5 exchange uses commercial, definitive implants cemented with high-dose antimicrobial PMMA. This method provides comfort and function, and patients are willing to accept longer exchange intervals as opposed to suffering with painful, loose, mal-fitting cement spacers, noting that the biofilm reformation risk is no less with an antimicrobial loaded cement spacer than definitive implants [38]. Lastly, for some patients, the 1.5 construct could function as a permanent terminal solution rather than a bridge to reimplantation. Three patients elected to retain their 1.5 construct at mean 4.2 years as they were satisfied with their arthroplasty.

The current results support future study of the antimicrobial brachytherapy concept, however not without limitations. First, focused intra-articular antimicrobial delivery with multimodal agents can impair local healing. ALCSB and antiseptic irrigants can impair fibroblast function, affecting healing. This can result in wound leaks requiring secondary debridement. Limiting intra-articular ALCSB volumes to 25–35 cc will mitigate drainage risk. Similarly, high-concentration antiseptic irrigants used with excessive dwell times can damage/kill host immune cells and fibroblasts, again risking wound leaks. Careful dwell time management with post-lavage saline rinse will help limit host toxicity [30]. During the burst release of antimicrobials from ALCSB and PMMA, systemic antimicrobial absorption can occur for up to 3–7 days depending on local antimicrobial load. This negates our efforts of eliminating systemic antimicrobials [36]. Doses of antimicrobials within ALCSB and PMMA require further study to arrive at the optimal balance that extinguishes microbial reserves yet mitigates systemic elution. A related limitation is that the ABP cannot yet uniformly minimize systemic antimicrobial exposure in fungal PJI. In this series, patients with fungal microbes required prolonged oral azole therapy based on sensitivity (16–24 weeks, or indefinite where a chronic extra-articular source such as pulmonary fungal infection was present). This departure from the short-course antimicrobial regimen reflects ongoing seeding from chronic extra-articular fungal sources and the ability of some fungal species to persist in a spore state. Fungal brachytherapy management therefore remains an evolving area requiring further investigation.

This study has additional limitations that warrant acknowledgement. First, the cohort was drawn from a single specialized referral service, introducing selection bias toward severely compromised hosts and limbs that is not representative of the general PJI population. Second, the absence of a concurrent control group precludes direct causal attribution of outcomes to the ABP, and historical comparisons should be interpreted cautiously. Third, the single-center design limits generalizability; multi-center validation will be required before broader application can be considered. The cohort is also small and underpowered, limiting interpretation of inferential statistics. However, this is the first report describing a tactical change in treatment for chronic PJI, with clinically relevant observations that warrant evaluation in larger comparative studies.

In our next iteration, we plan to further reduce oral antimicrobial duration, as the risk of host microbial dysbiosis from oral antimicrobials may be comparable to that associated with parenteral antimicrobials. Our planned reduction is in line with the emerging results of the SOLARIO trial [67]. Any further reduction in systemic antimicrobial exposure, apart from standard perioperative prophylaxis and extended therapy in chronic fungal infections, must be tested stepwise in prospective comparative studies before being recommended. Implementation of ABPs will require close multidisciplinary coordination among orthopedic surgeons, infectious disease specialists, and pharmacists, given the departure from conventional systemic antimicrobial regimens. Finally, as the 1.5 exchange concept grows in application, we suggest an addition to the MSIS Tier Outcome Reporting Tool: a Tier 1B category defined as 1.5 exchange with infection control and no continued antimicrobial therapy.

## 5. Conclusions

We introduce an antimicrobial brachytherapy protocol (ABP) for chronic PJI treatment and report preliminary outcomes in a small case series of TKA PJI, showing MSIS tier 1 results comparable to published two-stage protocols employing extended parenteral and oral antimicrobials. The ABP focuses therapy at the infection site, restricting systemic antimicrobials. It employs synergistic local antimicrobial modalities at resection using a 1.5 exchange technique to maintain comfort and function. This permits longer exchange intervals allowing individualized host and limb rejuvenation. These preliminary findings support further investigation of the ABP in larger, controlled studies before its role in clinical practice can be defined.

## Figures and Tables

**Table 2:** Microbes identified at resection *.

Identified Microbes
*Candida albicans*
*Coccidioides immitis*
*Cutibacterium acnes*
*Escherichia coli*
*Enterococcus faecium*
*Finegoldia magna*
*Lactobacillus kitasatonis*
*Proteus mirabilis*
*Prevotella melaninogenica*
*Serratia marcescens*
*Staphylococcus aureus*
*Staphylococcus aureus-methicillin resistant (mrsa)*
*Staphylococcus epidermidis*
*Staphylococcus hominis*
*Staphylococcus lugdunensis*
*Streptococcus mitis*
*Streptococcus oralis*
*Streptococcus agalactiae*
*Viridans group streptococci*

* Organisms identified on 2 or more preoperative aspirations and/or intraoperative cultures.

**Table 3:** Combined Host and Limb scoring; N = 25.

	Limb Score 1	Limb Score 2	Limb Score 3
Host Score A	1	0	2
Host Score B	2	6	11
Host Score C	0	1	2

**Table 4:** Tier 1 success by combined Host and Limb scoring; N = 25.

	Limb Score 1	Limb Score 2	Limb Score 3
Host Score A	100%	n/a	0%
Host Score B	100%	80%	63.3%
Host Score C	n/a	0%	50%

n/a = not applicable—no patients.

## Data Availability

All data pertinent to the current case has been presented as a part of this manuscript.
